# Genomic characterization of multidrug-resistant *Klebsiella pneumoniae* from an outbreak in Northeastern Brazil: mechanisms of virulence and resistance

**DOI:** 10.1007/s42770-026-01882-3

**Published:** 2026-02-25

**Authors:** Danillo Sales Rosa, Gabryel Bernardo Vieira de Lima, Henrique Da Silva Vieira, Flávia Figueira Aburjaile, Vasco Ariston de Carvalho Azevedo, Bertram Brenig, Mateus Matiuzzi da Costa

**Affiliations:** 1 Universidade Federal Rural de Pernambuco, Recife, 52171-900 Pernambuco Brazil; 2 Universidade Federal de Minas Gerais, Belo Horizonte, 31270-901 Minas Gerais Brazil; 3 Institute of Veterinary Medicine, University Göttingen, 37077 Göttingen, Germany; 4 Universidade Federal do Vale do São Francisco (UNIVASF), Petrolina, 56304-917 Pernambuco Brazil

**Keywords:** ESKAPE, MLST, Multiresistance, Resistome, Virulome

## Abstract

*Klebsiella pneumoniae* is an important nosocomial pathogen, considered a critical threat to public health, but its genomic data are scarce in Brazil. Therefore, the aim of this study was to identify the mechanisms of virulence and antimicrobial resistance and to evaluate the molecular epidemiology of *K. pneumoniae* isolates from an outbreak in a university hospital in Brazil. The DNA of the 25 isolates was sequenced, the functional annotation of the genome was performed in Prokka, for virulence and resistance analyses PanViTa was used, and for Multilocus Sequence Typing analyses pyMLST was used. The isolates presented several virulence genes such as those of the *fim* operon, *ecp* operon, and T6SS (identified in all isolates), which are related to adhesion, biofilm formation, and toxin delivery. Other predicted virulence genes involve nutritional/metabolic factors, effector delivery systems, and immune modulation, while resistance genes mainly involve efflux (AcrAB-TolC, KpnGH-TolC, OqxAB, and KpnEF), antibiotic target alteration, reduced permeability, and antibiotic inactivation. In addition, the resistance genes identified in *K. pneumoniae* isolates are related to a total of 28 antimicrobials from different classes. The sequence types (ST) 11, 273, 395, 5209 and 636 were identified, this being the first report of the ST636 in Brazil. Genomic analysis confirmed the presence of complex virulence and antimicrobial resistance mechanisms, highlighting the threat these pathogens pose and raising concerns about the introduction of ST636 into Brazil, emphasizing the complexity of local strains and the need for continuous surveillance.

**Supplementary Information:**

The online version contains supplementary material available at 10.1007/s42770-026-01882-3.

## Highlights

Framework of 110/82 virulence/resistance genes in *K. pneumoniae* isolates.

Resistance genes to 28 classes of antimicrobials were identified.

Adherence and nutritional factor genes are prevalent in the core genome.

Antibiotic efflux is the prevalent mechanism in the genomes.

First report of ST11, ST273, ST395, ST636 and ST5209 in Pernambuco, Brazil.

**Supplementary Information:**

The online version contains supplementary material available at 10.1007/s42770-026-01882-3.

## Introduction

The increase in cases of infections caused by bacteria resistant to multiple antibiotics is one of the biggest complications for public health, generally associated with a significantly higher cost [1] and culminating in a high mortality rate [2]. According to the World Health Organization [3], in 2023, one in six bacterial infections were related to antimicrobial-resistant pathogens, while data from the Disease Prevention and Control also warn of 35,000 deaths per year due to the same causes [4, 5]. In Brazil, Indonesia and Russia, it is estimated that infections caused by resistant microorganisms represent approximately 40–60% of all infectious diseases [6].

*Klebsiella pneumoniae*, in turn, makes up the group of important multidrug-resistant pathogenic bacteria of hospital nature, which is called “ESKAPE” (*Enterococcus faecium*, *Staphylococcus aureus*, *K. pneumoniae*, *Acinetobacter baumannii*, *Pseudomonas aeruginosa*, *Enterobacter* spp.) [7]. This pathogen is associated with infections such as pneumonia, pyogenic liver abscesses, necrotizing and soft tissue infections, bloodstream infections, meningitis, endophthalmitis, and urinary tract infections [7]. Given the severity of these infections and the lack of antimicrobials to treat them, *K. pneumoniae* resistant to carbapenems and third-generation cephalosporins are considered by the World Health Organization to be a critical threat to public health, making them a priority for the discovery of new antimicrobial agents [8].

It was predicted that resistant bacteria would cause approximately ten million deaths by the year 2050 [9]. However, in 2021, it was estimated that there were approximately 4.71 million deaths associated with bacterial resistance globally, of which ~ 1.3 million were directly attributed to the same phenomenon. Among the main pathogens related to deaths attributed to resistance are *S. aureus*, *K. pneumoniae*, *Streptococcus pneumoniae*, *A. baumannii*, *Escherichia coli* and *P. aeruginosa*, with at least 100,000 deaths. Additionally, regarding the pathogen-drug combination, carbapenem-resistant *K. pneumoniae* and 3rd-generation cephalosporin-resistant *K. pneumoniae* were responsible for almost 50,000 deaths attributable to resistance [10].

Clinical isolates of *K. pneumoniae* can use mechanisms such as active efflux, quorum sensing system and lipopolysaccharide production to form a rich and complex biofilm, making them even more virulent and resistant [11]. In this sense, biofilm production is considered one of the main causes of failure in the treatment of bacterial infections [12], as they act by positively regulating antimicrobial resistance and the expression of the efflux pump in *K. pneumoniae* [13]. During biofilm formation, efflux pumps assist in the excretion of molecules from the extracellular matrix and quorum sensing, which coordinates the efflux of harmful molecules and influences adhesion to surfaces and other cells [14]. Six families of proton-driven efflux pumps have been identified: the resistance-nodulation-cell division (RND) family, the proteobacterial antimicrobial compound efflux (PACE) family, the multidrug and toxic compound extrusion (MATE) family, the major facilitator superfamily (MFS), the minor multidrug resistance (SMR) family, and the p-aminobenzoyl-glutamate transporter (AbgT) family [15–17].

Therefore, understanding the virulence, resistance, transmission, and pathogenicity factors helps in the development of new antimicrobial options [18]. In this sense, high-throughput genomic analyses can reveal important insights into the population structure of *K. pneumoniae*, which may help to better understand how it evolves and spreads, causing diseases [19]. Therefore, by analyzing the genomic data of pathogenic microorganisms, it is possible to characterize, classify, and investigate the presence of genes related to resistance and virulence [20]. Furthermore, few studies with complete sequencing and identification of sequence types (STs) in Brazilian isolates have been identified [21, 22], which made the local epidemiological scenario unknown, hindering the understanding of which clinically important strains are being introduced into Brazilian territory and how they are spreading.

In view of this, it is of utmost importance to evaluate the dissemination of multidrug-resistant strains of *K. pneumoniae*, mainly within hospitals, in order to promote control measures for these pathogenic microorganisms. Therefore, the present study aimed to identify the mechanisms of virulence and resistance and to evaluate the molecular epidemiology of clinical isolates of *K. pneumoniae*.

## Materials and methods

### Bacterial isolates

Twenty-five multidrug-resistant *K. pneumoniae* (resistance to 2nd, 3rd, and 4th generation cephalosporins and with the vast majority resistant to carbapenems) isolated from colonized and/or infected patients admitted to the University Hospital of the Federal University of Vale do São Francisco (HU-UNIVASF), Petrolina, Pernambuco, Brazil, from July to November 2021 were used [23]. These isolates came from blood culture (2/25), rectal swab (8/25), surveillance culture (2/25), tracheal secretion (10/25), and urine culture (3/25). Additionally, information on the origin, initial identification method, and phenotypic data on resistance and biofilm formation capacity of these isolates is available in Rosa et al. [23]. These isolates are registered in the National Genetic Heritage Management System (SisGen, No. A01C760) and their acquisition was approved by the ethics committee of the University of the Campanha Region (URCAMP) – No. 5.079.225.

### DNA extraction

*K. pneumoniae* isolates were cultured in tryptic soy agar (TSA, Kasvi, São José dos Pinhais, Brazil) at 37 °C for 24 h and subjected to the genomic DNA extraction methodology, according to Regitano [24]. DNA quality was assessed in a 1% agarose gel and quantification in a NanoDrop One/OneC spectrophotometer (Thermo Fisher Scientific, Waltham, United States).

### Sequencing and quality control

Sequencing was performed with the support of the Omics Sciences Network (RECOM) using the Hiseq 2500 platform (Illumina, San Diego, United States) with the 150 bp Truseq paired-end library.

The samples acquired through sequencing were subjected to initial quality analysis in FastQc v0.12.1 [25], where the presence of adapters, %GC, Phred, among others, was evaluated. From this, those that presented low-quality reads or adapters were trimmed before assembly.

Two different trimming methodologies were used individually on the samples, to verify which would be more effective. Trimmomatic v0.39 [26] was used to remove low-quality reads and adapters. The AdapterRemoval v2.3.2 tool [27] was also used.

### Assembly and quality control

To assemble all sequences, Unicycler v0.4.8 [28] was used, establishing a minimum size limit of 200 base pairs for a contig, a standard established by the National Center for Biotechnology Information (NCBI) for deposit. First, to analyze the quality of the assemblies, Quast v5.0.2 [29] verified the quality patterns among the genomes, with a greater focus on N50, L50, the total sequence size and number of contigs, to decide which trimmed assembly to use. Subsequently, the BUSCO v5.4.7 (Benchmarking Universal Single-Copy Orthologs) [30] was performed, with all isolates and those subsequently acquired by NCBI, to investigate genome completeness. Finally, the analysis regarding bias in the samples was carried out, using CheckM v1.2.2 [31].

### Taxonomy and functional annotatione

To perform the TCS (Tetra Correlation Search) analysis, the JSpecies platform (available at <https://jspecies.ribohost.com/jspeciesws/%3E) was used. The genomes were submitted to obtain the Z-score result for the species in the platform’s database.

The functional annotation of the genome was carried using Prokka v 1.14.6 (Prokaryotic Genome Annotation) [32], thus enabling the following analyses.

### Analysis of Pan-Virulome and Pan-Resistome genes

To identify genes related to resistance and virulence of the isolates, PanViTa v1.1.5 [22] was used, with 80% identity and 80% coverage, to perform a search in two of the three databases, CARD (Comprehensive Antibiotic Resistance Database) [33] for resistance genes and VFDB (Virulence Factor DataBase) for virulence genes [34].

### In Silico multilocus sequence typing (MLST) and core genome MLST (cgMLST) analysis

Using the pyMLST v2.1.6 software [35], MLST and cgMLST analyses were performed. To identify the STs by MLST, the seven housekeeping genes of *K. pneumoniae* were used, and the data generated were subsequently used in PHYLOViZ v 2 [36] to construct the minimum sparsity tree. For cgMLST, information from RIDOM (Ribosimal Differentiation of Medical Micro-organisms Database) (https://www.cgmlst.org/ncs/schema/2187931/) was imported to, together with the genomes of the present study, compose the DATABASE of the core genome of *K. pneumoniae*. Based on the alignment of these sequences, the distance matrix and Multiple Sequence Alignment (MSA) were generated. Subsequently, the MSA data were converted to Newick format using GrapeTree [37], for later visualization in Evolview v 2 [38].

Orthology analysis.

A phylogenetic dendrogram was created using the genomes of the 25 *K. pneumoniae* isolates from this study and 347 complete *K. pneumoniae* genomes of human origin available at NCBI until April 2025 (as exclusion criteria, those that presented missing information such as location, disease and/or host were removed), which were previously submitted together in the PanViTa analysis for comparative purposes. The *E. coli* K12 (Genome assembly: ASM584v2) were also integrated in the data as an outgroup, for the orthology analysis only. To create the phylogenetic dendrogram, single-copy orthologs common to all genomes were deduced by OrthoFinder v. 2.5.5, using Mafft as the alignment tool to perform the Multiple Sequence Alignment (MSA), with the Fasttree to the inference of the tree. Subsequently, with Iqtree the Maximum Likelihood were performed using 1000 replicates to the bootstrap analysis, for more reliability of the tree. Furthermore, the tree was visualized in Interactive Tree of Life (ITOL) v7.2.

## Results

### Sequencing and assembly quality

Based on the quality analyses, Quast demonstrated that the genomes ranged from 5,058,188 to 5,895,722 base pairs, in accordance with the average size expected for the species in the NCBI (5.7 Mb). The genome size of each isolate, as well as the quality metrics, can be seen in Table 1. Based on the NCBI, the *K. pneumoniae* genome has around 5,779 coding sequences. In the genomes annotated in this study, it was possible to observe the presence of 4,647 to 5,591 coding sequences, in addition to 80 to 87 non-coding sequences, which include rRNA, repeat region, tRNA and tmRNA. The assembled sequences were deposited in the NCBI under bioproject PRJNA1161663.

BUSCO report revealed that all genomes had genes missing from the database (4 or 5 per genome), in addition to 2 duplicated genes and 1 fragmented gene in all genomes (Supplementary Figure S1)

**Table 1:** Quality metrics and characterization of the sequenced *Klebsiella pneumoniae* isolates, from the university hospital of the federal university of Vale do São Francisco, Petrolina - PE

Isolate	Contigs	Size (bp)	N50	L50	CDS	Genes	rRNA	Repeat region	tRNA	tmRNA	Coverage
KLPN_5816	104	5,778,190	234,242	9	5458	5540	3	-	78	1	122,598
KLPN_3042	97	5,760,157	229,274	9	5437	5519	3	1	78	1	85,103
KLPN_9944	106	5,775,826	214,402	10	5455	5537	3	1	78	1	92,241
KLPN_5984	100	5,765,899	229,274	10	5452	5534	3	1	78	1	121,427
KLPN_253	91	5,883,814	218,179	9	5573	5659	4	-	81	1	160,546
KLPN_3233	74	5,622,372	388,545	4	5245	5327	3	-	78	1	145,667
KLPN_6002	92	5,883,108	221,822	8	5571	5658	5	-	81	1	111,514
KLPN_3125	96	5,883,150	212,807	9	5567	5654	5	-	81	1	118,242
KLPN_5773	126	5,807,322	172,503	11	5504	5587	3	1	79	1	190,218
KLPN_5794	86	5,624,689	214,402	10	5301	5383	3	1	78	1	180,050
KLPN_5799	106	5,775,923	208,502	10	5459	5541	3	1	78	1	129,828
KLPN_3065	107	5,766,802	208,065	10	5445	5527	3	1	78	1	77,501
KLPN_5893	110	5,776,711	164,397	11	5454	5536	3	1	78	1	147,101
KLPN_5887	27	5,058,188	376,585	5	4647	4730	7	-	75	1	62,929
KLPN_9322	109	5,774,817	164,397	11	5458	5540	3	1	78	1	56,532
KLPN_9338	110	5,777,643	164,397	11	5457	5539	3	1	78	1	134,112
KLPN_5994	98	5,895,722	199,455	9	5591	5678	5	-	81	1	63,438
KLPN_3272	108	5,756,968	207,601	10	5437	5519	3	1	78	1	112,782
KLPN_3271	108	5,777,621	214,402	10	5457	5539	3	1	78	1	174,582
KLPN_3126	92	5,881,792	221,822	8	5573	5660	5	-	81	1	147,885
KLPN_9273	74	5,473,923	306,227	6	5133	5215	3	-	78	1	118,489
KLPN_3139	101	5,880,283	217,632	8	5573	5660	5	-	81	1	62,856
KLPN_3174	90	5,827,699	229,951	8	5501	5586	5	-	79	1	176,676
KLPN_6001	97	5,812,988	229,055	8	5499	5586	5	-	81	1	59,587
KLPN_5825	90	5,833,226	247,216	8	5586	5666	3	-	77	-	104,069

### Pan-Virulome and Pan-Resistome

For the 25 genomes, 110 virulence-related genes (Supplementary Figure S2) and 82 antimicrobial resistance genes (Supplementary Figure S3) were predicted, which are described in Supplementary Tables 1 and 2, respectively.

**Fig. 1:**
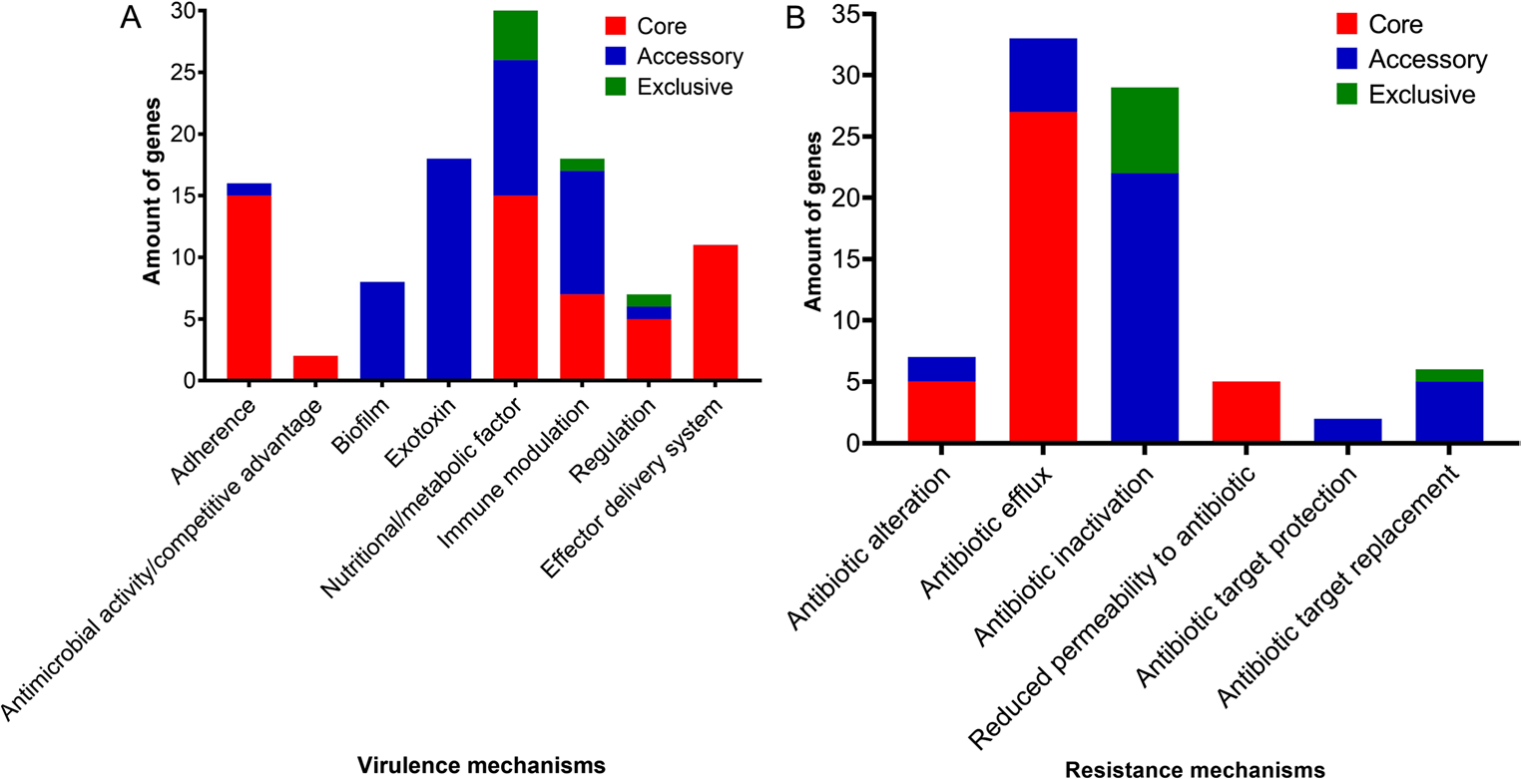
Virulence and resistance mechanisms identified in the genomes of *Klebsiella pneumoniae* isolates from the University Hospital of the Federal University of Vale do São Francisco, Petrolina – PE. Virulence (**A**) and antimicrobial resistance (**B**) mechanisms. Red bar (core genes), blue (accessory genes) and green (genes exclusive to the one genome)

The genome KLPN_3174 had the highest number of virulence genes (95), while KLPN_3233 had the lowest number (75). The genomes KLPN_3126, KLPN_3125, KLPN_6001, KLPN_253, KLPN_3139, KLPN_6002 and KLPN_5994 presented 84 genes and the others, 76 genes. Regarding antimicrobial resistance genes, the genomes KLPN_5887, KLPN_6001 and KLPN_9273 presented the smallest amounts with 39, 42 and 44 genes, respectively, while the others presented 49 to 60 genes. In respect of the functionality of the predicted genes, Fig. 1 shows the variability of mechanisms involving virulence (Fig. 1A) and antimicrobial resistance (Fig. 1B), which can be divided into core genes (shared by all genomes used), accessory genes (present in more than one of the genomes used, but not in all) and exclusive genes (present in only one of the genomes). The number of genes predicted for each virulence mechanism can be observed below: 55 core genes [adherence (15), nutritional/metabolic factor (15), effector delivery system (11), immune modulation (7), regulation (5) and antimicrobial activity/competitive advantage (2)]; 49 accessory genes [exotoxin (18), nutritional/metabolic factor (11), immune modulation (10), biofilm (8), adherence (1) and regulation (1)]; and 6 unique genes [nutritional/metabolic factor (4), immune modulation (1), and regulation (1)].

As for the mechanisms of antimicrobial resistance, the number of predicted genes was: 37 core genes [antibiotic efflux (27, including members of the RND, MFS, ATP-binding cassette (ABC), SMR, and MATE families), antibiotic target switching (5), and reduced antibiotic permeability (5)]; 37 accessory genes [antibiotic inactivation (22), antibiotic efflux (6), antibiotic target substitution (5), antibiotic target switching (2), and antibiotic target protection (2)]; and 8 unique genes [antibiotic inactivation (7) and antibiotic target substitution (1)].

**Fig. 2:**
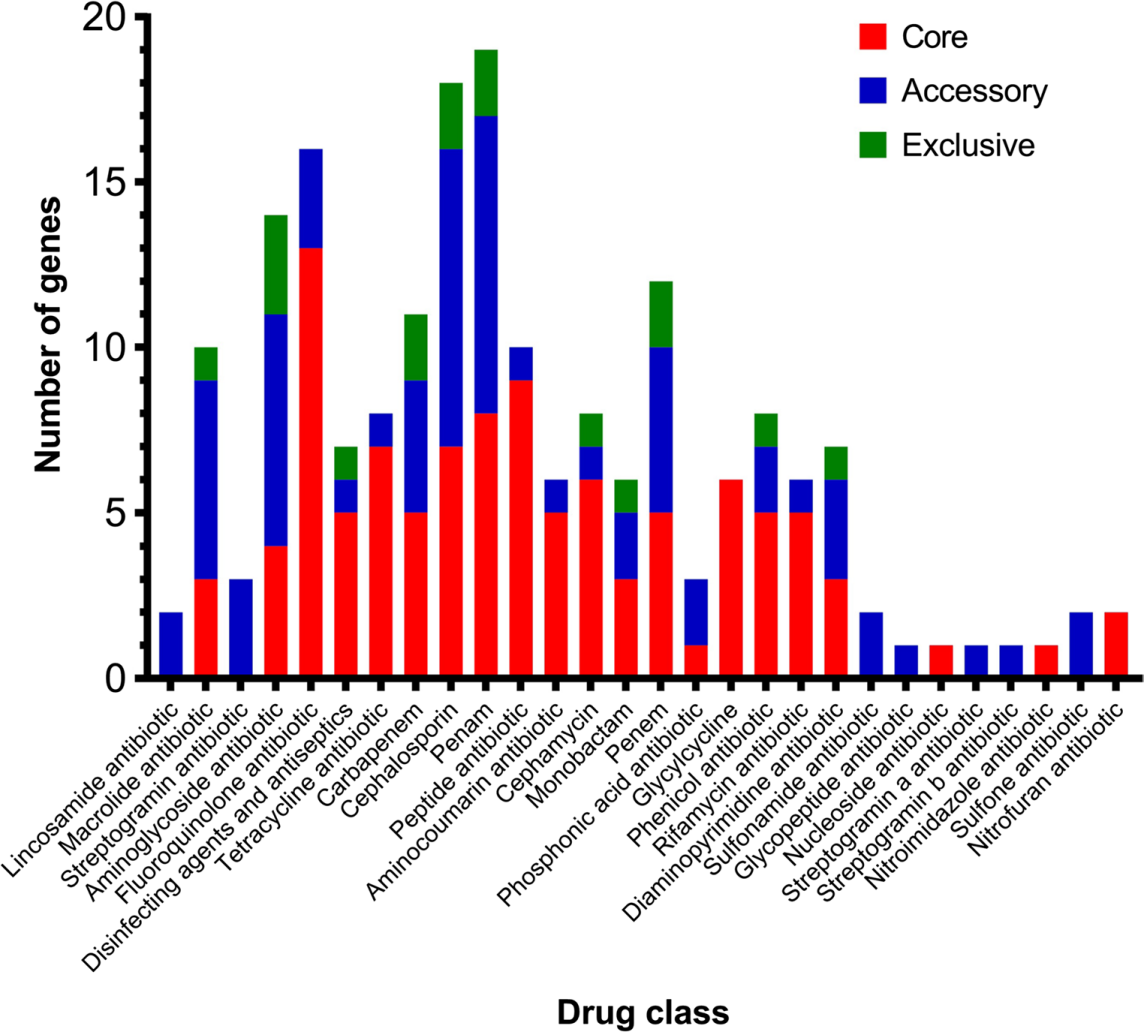
Classes of antimicrobials for which resistance genes were identified in the genomes of *Klebsiella pneumoniae* isolates from the University Hospital of the Universidade Federal do Vale do São Francisco, Petrolina – PE. Red bar (core genes), blue (accessory genes) and green (exclusive genes)

As can be seen in Fig. 2, the resistance genes identified in the *K. pneumoniae* isolates are related to a total of 28 classes of antimicrobials, which are part of the: core genes [fluoroquinolones (13), peptides (9), penams (8), cephalosporins (7), tetracyclines (7), cephamycins (6), glycoglycins (6), disinfectants (5), aminocoumarins (5), carbapenems (5), penems (5), rifamycins (5), aminoglycosides (4), diaminopyrimidines (3), macrolides (3), monobactams (3), phosphonic acids (1), phenicols (1), nucleosides (1)].

Regarding the classes that are part of the accessory genes, the following were observed: cephalosporins (9), penams (9), aminoglycosides (7), macrolides (6), penem (5), carbapenems (4), streptogramins (3), phosphonic acids (2), phenicols (2), fluoroquinolones (3), lincosamides (2), monobactams (2), sulfonamides (2), sulfones (2), disinfectant agents (1), aminocoumarins (1), cephamycin (1), peptides (1), diaminopyrimidines (1), streptogramins a (1), streptogramins b (1), glycopeptides (1), rifamycins (1), tetracyclines (1)]; and exclusive genes [aminoglycosides (3), carbapenems (2), cephalosporins (2), penams (2), penems (2), disinfectants (1), cephamycin (1), diaminopytimidines (1), phenicols (1), macrolides (1), monobactams (1).

### MLST and CgMLST

**Fig. 3:**
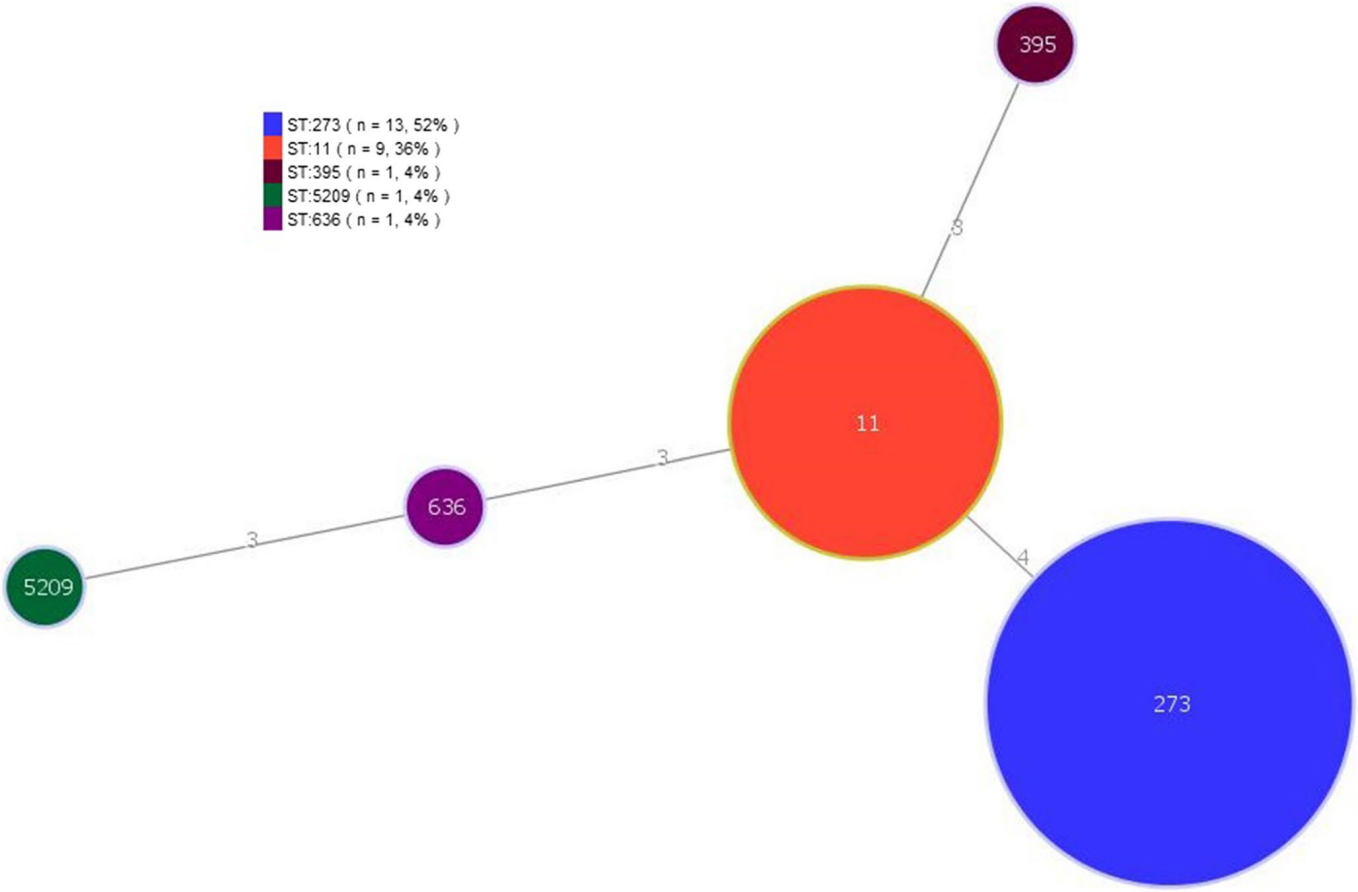
Minimum sparsity tree based on Multilocus Sequence Typing of 25 *Klebsiella pneumoniae* isolates, generated in PHYLOiZ

**Fig. 4:**
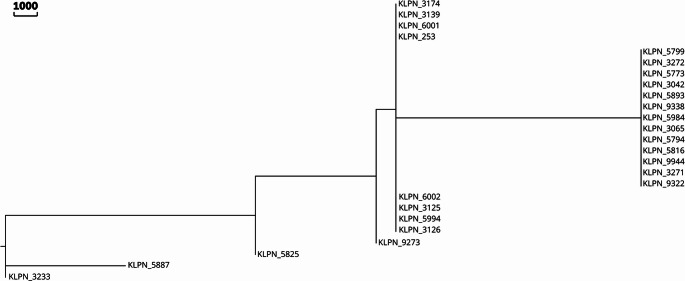
Tree, generated in GrapeTree and visualized in Evolview, based on Multiple Sequence Alignment (MSA) data from the Multilocus Sequence Typing core of *Klebsiella pneumoniae*

MLST analyses revealed that the genomes of the isolates were classified into five STs: ST11 (*n* = 9; 36%); ST273 (*n* = 13; 52%); ST 395 (*n* = 1; 4%); ST636 (*n* = 1; 4%) and ST5209 (*n* = 1; 4%). Figure 3 shows the MLST-based minimum sparsity tree of *K. pneumoniae* isolates, and information on the alleles and ST of each isolate can be found in Supplementary Table 3. Figure 4 shows the tree generated based on the MSA data of the cgMLST of *K. pneumoniae*, in which 52% of the genomes are grouped in the same clade.

**Fig. 5:**
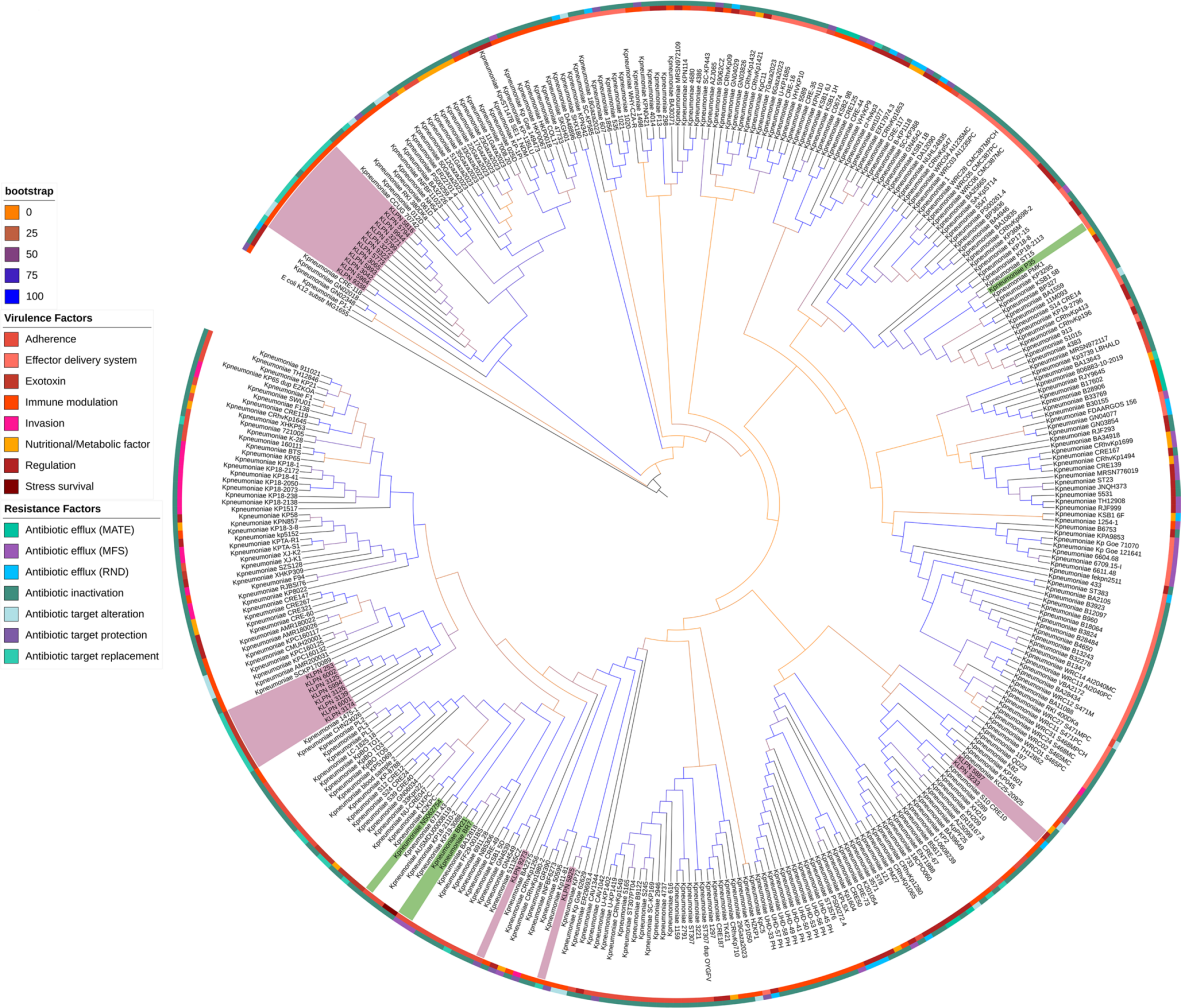
Tree, generated in OrthoFinder and visualized in Interactive Tree of Life (ITOL), based on Multiple Sequence Alignment (MSA) data of *Klebsiella pneumoniae*. The genomes of the isolates in this study are highlighted in pink, while the publicly available genomes of other Brazilian isolates are highlighted in green

As can be seen in Fig. 5, after aligning the genomes of this study with those available at NCBI, the same group of isolates (52%) remained in the same clade. The grouping of the remaining samples also remained similar, differing only in the penultimate branches of the cgMLST tree (Fig. 4). In this clade, the genomes were separated into two clades with four isolates, but based on the complete genome (Fig. 5), all eight were grouped into the same clade. However, in both, the isolates share a common ancestor.

It can be observed that in general the most prevalent resistance factor in *K. pneumoniae* is antibiotic inactivation (255/372), while the most prevalent virulence factors are immune modulation (95/372), adhesion (77/372) and effector delivery system (74/372). The four genomes of Brazilian isolates show a prevalence of resistance and virulence mechanisms similar to those identified in the isolates of the present study.

Furthermore, it can be observed that the predominant virulence factors in the isolates of the present study are immune modulation (15/25) and regulation (9/25), while the predominant resistance factor is antibiotic target replacement (19/25). Additionally, most of the isolates in the present study showed a phylogenetic relationship with *K. pneumoniae* strains of Asian origin.

## Discussion

Genomic data in Brazil is scarce, and the first report of a multidrug-resistant *K. pneumoniae* genome from the northeastern region of the country occurred in 2021 [39]. This bacterial sample was isolated in 2016 from bronchoalveolar lavage of a patient with respiratory symptoms in an intensive care unit in the state of Sergipe, and the authors of the study draw attention to the need for genomic surveillance studies in Brazil. Therefore, in the present study, we supplement this with genomic data from another 25 multidrug-resistant *K. pneumoniae* isolates from Northeast Brazil, this time from the state of Pernambuco. Several virulence genes have been identified in these isolates, including those related to the production of fimbriae, which are adhesins that aid in bacterial attachment to biotic and abiotic surfaces, in the invasion of epithelial cells and in the formation of biofilms [40, 41]. All genomes shared most of the genes of the *fim* operon (*fimABCDEFGHIK*), which is related to the production of type 1 fimbriae [42]. Dan et al. [43]. observed the presence of the *fimH* gene in most (91.7%, 88/96) of *K. pneumoniae* strains from various samples (sputum, urine, blood, secretions, thoraco-abdominal fluid and bile), obtained from patients at a university hospital in China (The Affiliated Chaohu Hospital of Anhui Medical University) between 2018 and 2019, and these strains showed the ability to form biofilms in vitro. The homologue of the *ecp* operon (*ecpABCDE*) was also found in the isolates of the present study. It is responsible for the expression of *E. coli* common pilus (ECP), which, in *K. pneumoniae*, contributes to cell adhesion, biofilm formation and colonization of various niches [44]. Additionally, the *mrk* operon (*mrkABCDF*), responsible for the expression of type 3 fimbriae [42], was present in most genomes (68%, 17/25), here classified in the biofilm mechanism. The *cpxA* gene was present in all genomes; however, it is part of a two-component system, CpxAR, which acts in the negative regulation of the expression of type 3 fimbriae [45], but *cpxR* was not present. In addition, the *crp* gene was present, which is a regulator in response to glucose and oxygen availability [46], and indirectly regulates the expression of type 3 fimbriae through the c-di-GMP signal pathway [47]. Therefore, glucose and the catabolite repressor protein (CRP) can affect the resistance of bacteria to polymyxins [48]. *K. pneumoniae* is known to be capable of producing several types of pili, which can act together to colonize the host [44]. Consistent with this, the isolates in the present study demonstrated, in previous assays [23], the ability to form biofilms in different proportions on the surface of 96-well microplates, demonstrating the potential of these isolates to cause infections and persist in the hospital environment.

Regarding antimicrobial activity/competitive advantage, efflux system genes from the RND, MFS, ABC, SMR, and MATE families were identified. The *acrA*, *acrB*, and *tolC* genes were present in all genomes and express a multidrug efflux system from the RND family, which confers resistance to quinolones, such as ciprofloxacin, and decreased susceptibility to carbapenems [49], corroborating the phenotypic resistance data previously identified in these isolates, which were all resistant to levofloxacin e imipenem (92%, 23/25), meropenem, ertapenem and ciprofloxacin (96%, 24/25) [23]. Genomic characterization has also contributed to several Brazilian studies, such as the identification of carbapenem resistance in a study with a (1/1) clinical strain of *K. pneumoniae* that was responsible for a large outbreak in a university hospital in Paraná in 2009 and was obtained from blood culture of a patient in an intensive care unit [50]; as well as the identification of resistance and discovery of new mutations in genes related to colistin resistance in ten resistant *K. pneumoniae* isolates, originating from patients from two hospitals in Rio de Janeiro in 2016 [51]. A secondary periplasmic transporter (AcrA), an internal membrane (AcrB), and a multidrug efflux pump system known as AcrAB-TolC [52] are present. Furthermore, increased efflux through this system is one of the main factors contributing to the tolerance of *K. pneumoniae* to biocides such as chlorhexidine [53]. Additionally, the *acrF* and *acrD* genes, which are believed to function with *acrA* and *tolC*, encoded at different locations in the genome [54, 55], were also present in the genomes of this study. The *marA* and *ramA* genes were identified in all genomes of the present study and these positively control the AcrAB-TolC efflux pump [56–58]. The MFS gene, *emrR* from *E. coli*, has been identified and encodes a repressor of the EmrAB-TolC efflux pump [59], but the other genes are missing. However, the *kpnG* and *kpnH* genes have been identified, which encode the KpnGH-TolC efflux pump, also dependent on TolC and homologous to EmrAB-TolC, whose inactivation improves susceptibility to azithromycin, ceftazidime, ciprofloxacin, ertapenem, erythromycin, gentamicin, imipenem, ticarcillin, norfloxacin, polymyxin-B, piperacillin, spectinomycin, tobramycin and streptomycin [60].

The *hns* gene, of the histone-like nucleoid structuring protein (H-NS), was present and is responsible for repressing the expression of some TolC-dependent efflux pump genes (acrEF-tolC and mdtEF-tolC), and whose deletion confers multidrug resistance in *acrAB*-*tolC*-deficient strain [61]. However, the genomes also presented the LeuO gene, which is a global regulator of multiple loci, including genes related to stress response and pathogenicity of Enterobacteriaceae, which competes with H-NS, limiting the repression caused by it [62]. The mdtB and mdtC genes were present in the genomes (100%, 25/25), while the mdtA gene was identified in 56% (14/25) of them. However, in previous phenotypic analyses [23] with the same isolates, a low resistance rate (28%, 7/25) to tigecycline was observed. The expression of MdtABC-TolC, which reduces susceptibility to tigecycline, eravacycline and omadacycline and exhibits heteroresistance to tigecycline, can be modulated by several regulatory systems such as BaeSR and CpxRA [63], which are absent or incomplete in the genomes of the present study; but may also involve small redulatory RNAs such as sRNA-120 from *K. pneumoiae*, which inhibits the expression of MdtABC-TolC, among other unknown regulators [63]. In addition, together with *acrB* and *acrD*, they are involved in enterobactin efflux [64]. The *mdtG* and *mdtH* genes have been identified, are members of the MFS and function in the efflux of chloramphenicol-doxorubicin, norfloxacin, tetracycline, novobiocin, and nalidixic acid [65]. The *mdtK* gene encodes an efflux pump for the extrusion of acriflavine, doxorubicin, norfloxacin, and dipeptides [66, 67]. The *oqxA* and *oqxB* genes, which encode the OqxAB efflux pump, are related to fluoroquinolone resistance in *K. pneumoniae*, and related to extended-spectrum beta-lactamase (ESBL)-producing and carbapenemase-producing isolates, as well as glycylcyclines such as tigecycline [68, 69]. The *rsmA* gene belongs to RND and regulates quorum sensing, but when mutated it is linked to increased biofilm production, elastase and antibiotic resistance [70].

The MFS gene *mdfA* was also found in all genomes and is known to confer resistance to fluoroquinolones, erythromycin, chloramphenicol, and aminoglycosides [71], however, none of the isolates showed resistance to amikacin [23]. The *kdpD* and *kdpE* genes together encode the KdpD/KdpE two-component system, which regulates the potassium ion (K) pump operon Kdp-ATPase kdpFABC [72], and although *kpdD* is absent in the isolates of the present study, it has been reported that, in *S. aureus*, *kdpE* regulates the transcription of several virulence targets, such as those related to microbicide exposure and quorum sensing [73]. The *kpnE* and *kpnF* genes of the KpnEF efflux pump, SMR family, were present and confer resistance to several dyes, detergents and antimicrobial compounds such as benzalkonium chloride, ceftriaxone, chlorhexidine, acriflavine, cefepime, colistin, erythromycin, streptomycin, rifampicin, triclosan and tetracycline [74]. *Yojl* expresses an ABC exporter that aids in resistance to the antibiotic peptide microcin J25, maintaining its intracellular concentration below toxic levels, and this extrusion is assisted by TolC [75]. The *bacA* gene confers resistance to antibiotic peptide via molecular bypass [76].

In addition, the Type VI Secretion System (*T6SS*) was identified as being related to the effector delivery system, which confers a competitive advantage by allowing the delivery of toxins to neighboring cells, confers the ability to form biofilms and acquire resistance to antimicrobials [77]. Nutritional/metabolic factors were also observed in these genomes, such as several genes related to the production of siderophores, including enterobactin, yersiniabactin, aerobactin and salmochelin, which are related to the capture and competition for free iron present in the host, configuring an important virulence factor [78]. Similar genes, related to siderophore production, have been widely identified in other Brazilian isolates, mainly from the state of São Paulo [79], however, they were not identified in the isolate from the state of Sergipe [39]. Other genes related to altering the antibiotic target and reducing permeability are present in all genomes. The upregulated *arnT* gene, among other genes, contributes to colistin resistance in *K. pneumoniae* [80]. The *eptB*, *pmrF* and *ugd* genes were core genes and are described to be associated with polymyxin resistance through modifications in lipopolysaccharide (LPS) [81] and 60% of these isolates showed resistance to polymyxin [23]. The *kpne*_*ompK37* gene produces a porin with a smaller pore than the OmpK35 and OmpK36 porins and is related to lower sensitivity to beta-lactams [82]. The *mdtQ* gene encodes a multidrug-resistant outer membrane protein [83].

Similarly, regarding immune modulation, the genomes share a series of genes related to capsule formation, such as *galF*, associated with capsular polysaccharide (CPS) biosynthesis [84], and *rmpA*, a transcriptional regulator that promotes increased virulence of *K. pneumoniae* by increasing promoter activity to activate CPS production [85]. The *rmpA*/*A2* gene has been widely identified in other Brazilian isolates, which are associated with a hypermucoviscosity phenotype [79], but it was also not present in the isolate from the state of Sergipe [39]. *GndA* was also present, a gene within the cps locus related to serotype K2 and capsule formation [86]. Furthermore, other genes related to LPS production were identified, which, in addition to being one of the factors that make bacteria resistant to antibiotics, is an important virulence factor that triggers the host immune response [87]. For example, *kdsA*, which is related to the synthesis of Kdo, which binds lipid A and core oligosaccharides [88], its product (KdsA) is considered an important putative target for the identification of new drug targets against *K. pneumoniae* [89]. All genomes presented the *lptD* gene, which is related to the transport of LPS to the outer leaflet of the outer membrane, through an ABC transporter comprising LptA, LptB2FGC and LptDE [90]; however, the other genes were not identified. The genomes also presented *msbA*, which expresses an inner membrane protein that transports LPS from the inner leaflet to the periplasmic face of the inner membrane [91]. Additionally, the presence of *ompA* demonstrates the potential to produce OmpA, which induces the production of pro-inflammatory cells and affects host cell viability, causing apoptosis and pyroptosis [92]. The *ugd* gene product, UDP-glucose-6-dehydrogenase, is related to colistin resistance [93].

For *K. pneumoniae*, the clonal complex (CC) CC258 is reported as a significant cause of outbreaks, responsible for 68% of occurrences, and this includes ST258, ST11, ST512 [94]. ST11, one of those identified in the present study, is recurrently identified in Brazil and represents a high risk associated with the dissemination of carbapenemase-producing *K. pneumoniae* (KPC), including hypervirulent (hv) and hypermucoviscous (hm) [42]. In Brazil, in a study carried out with 46 sequenced *K. pneumoniae* samples, observed that ST11 (*n* = 16) was the most prevalent ST (34.78%), which was found in the states of Bahia (4/16), Ceará (2/16), Espirito Santo (6/16), Minas Gerais (2/16) and Tocantins (2/16), five of seven Brazilian states sampled [95]. In the same study, ST273 (*n* = 1/46, 2.17%) were identified in Bahia and ST395 (*n* = 1/46, 2.17%) in Ceará. In unpublished data, obtained from www.onehealthbr.com, reports of ST273 were observed in Brazil in a sample from Paraíba in 2018 (SAMN17014748) and São Paulo in 2016 (PPHO00000000.1). ST273 is part of CC147, together with ST147 and ST392 [96] and has also been reported in Central-West Brazil, as well as ST11 [97]. We identified ST395 (KLPN_5825) among the samples and this is a less characterized lineage, but it presents significant risks because it is multidrug resistant (MDR) and presents ESBL, in addition to carbapenemases and 16 S rRNA methyltransferases, responsible for conferring resistance to pan-aminoglycosides [98]. ST395 was also reported in Brazil, in Paraíba state in 2019 (SAMN17036468). ST5209 was first identified by Nakamura-Silva et al. [21]., in a sample of *K. pneumoniae* isolated from a tracheal aspirate of a patient at a hospital in Manaus, Amazonas, Brazil, in 2014. The new description was due to the new tonB allele (705). ST636 is poorly described in the literature, but has been reported in Croatia in clinical isolates collected between 2013 and 2014 [99] and in Norway in samples obtained between 2017 and 2018 [100], but had not been reported in Brazil to date. Additionally, this is the first report of these STs in Pernambuco, Brazil.

Based on phylogenetic analyses, the sequenced isolates show the greatest similarity to isolates from outside Brazil. Most of the genomes in this study are related to *K. pneumoniae* CCUG_70742 (GenBank accession no. GCF_003194285.1), isolated from a case of acute cystitis in Gothenburg, Sweden. The second largest cluster is related to *K. pneumoniae* SCKP170089 (GenBank accession no. GCF_032833975.1), originating from a bloodstream infection in Changhua, Taiwan. The genomes KLPN 5887 and KLPN 3233 are most closely related to *K. pneumoniae* S10 CRE10 (GenBank accession no. GCF_027595585.1), isolated from a case of urinary tract infection related to direct catheterization in Durham, North Carolina, USA. The genome KLPN_5825 is related to *K. pneumoniae* KP72 (GenBank accession no. GCF_024396895.1), isolated from a urinary tract infection in Kunming, China. Finally, the genome KLPN 9273 is related to *K. pneumoniae* 51135CZ (GenBank accession no. GCF_020695665.1), isolated from urine in a nonspecific fever in the Zlin Region, Czech Republic. The presence of more than one cluster suggests that the outbreak was not caused by a single bacterial lineage that spread, but rather by multiple different lineages or clones of *K. pneumoniae* that coexisted and caused infections at the same time and location. This highlights the complexity of the epidemiological situation in the hospital. Furthermore, the unique strains not clustered with the others likely represent sporadic or isolated cases that are not directly linked to the main transmission events within the outbreak, or that were introduced from sources outside the main outbreak.

## Conclusion

Genomic analysis of *K. pneumoniae* isolates identified several virulence and multidrug resistance genes, highlighting the complexity of the strains. The identified gene scaffold demonstrates the capability of these isolates to cause infections and persist in the hospital environment due to their diversity of genes to resist both disinfectants and antibiotic therapy. These data are fundamental for informing treatment choices and can assist in the development of more effective strategies to prevent and control the spread of *K. pneumoniae*. The isolates in this study are related to important CCs and some recently described STs in Brazil. Furthermore, this is the first report of ST636 in Brazilian territory, as well as STs 11, 273, 395, 636 and 5209 in the state of Pernambuco, Brazil. Additionally, the phylogeny of the isolates suggests that the outbreak was caused by multiple different lineages or clones, but that there were unique isolates that were not associated with the main outbreak. Together, these data emphasize the importance of continuous genomic surveillance to monitor the evolution and spread of these pathogens within Brazilian hospital environments, where genomic data are still scarce.

## Supplementary Information

Below is the link to the electronic supplementary material.

Supplementary Material 1 (JPEG 432 KB)

Supplementary Material 2 (JPEG 472 KB)

Supplementary Material 3 (JPEG 364 KB)

Supplementary Material 4 (DOCX 56.0 KB)

## Data Availability

All data supporting the findings of this study are available within the paper and its Supplementary Information.
