# Supplementary figures and images for: Genomic characterization of multidrug-resistant Klebsiella pneumoniae from an outbreak in Northeastern Brazil: mechanisms of virulence and resistance
Source: Braz J Microbiol. 2026 Feb 25;57(1):62. doi: 10.1007/s42770-026-01882-3 (PMC12936292; doi:10.1007/s42770-026-01882-3)

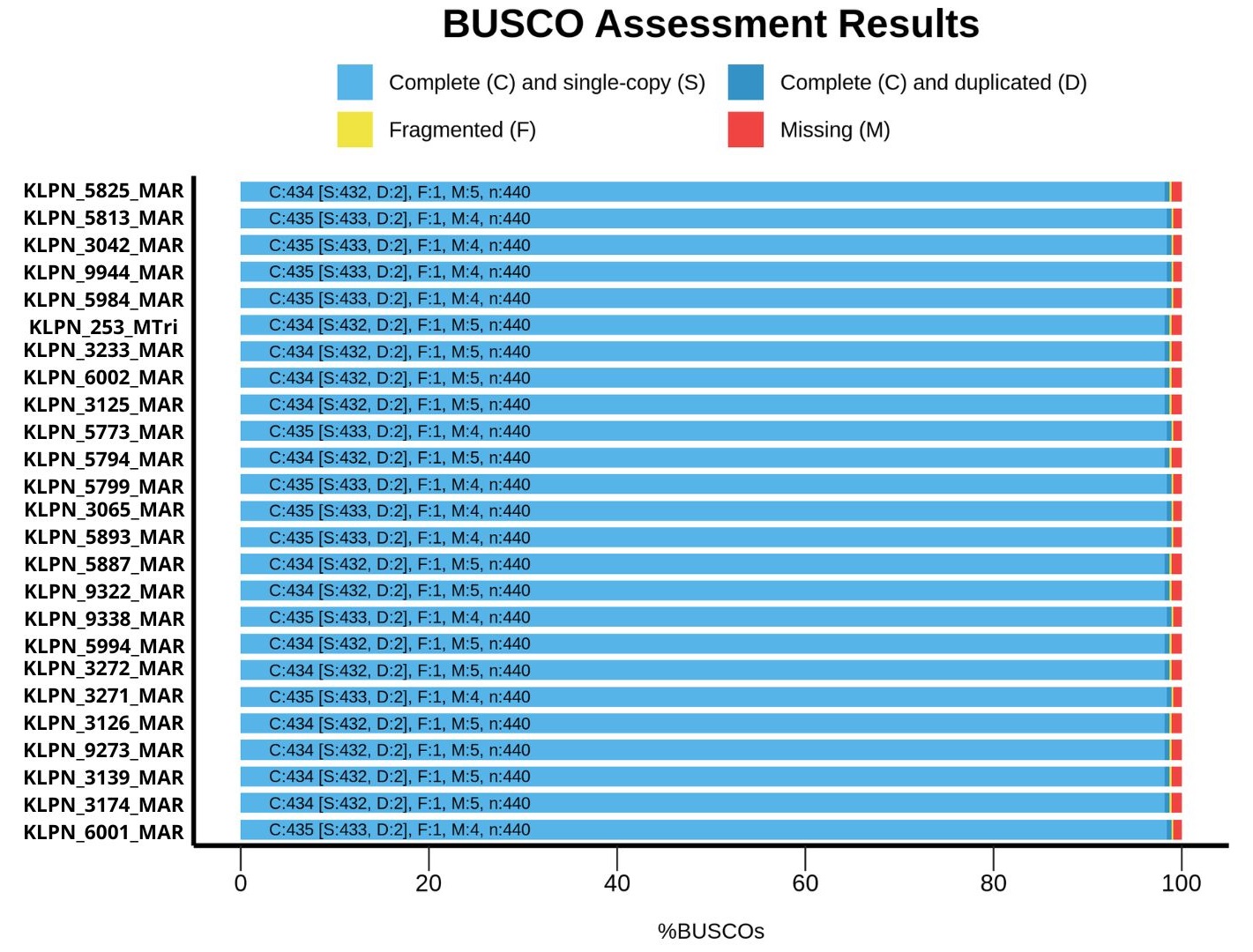

Supplement: Supplementary file 1 — Supplementary Material 1 (JPEG 432 KB) [file 42770_2026_1882_MOESM1_ESM.jpg]

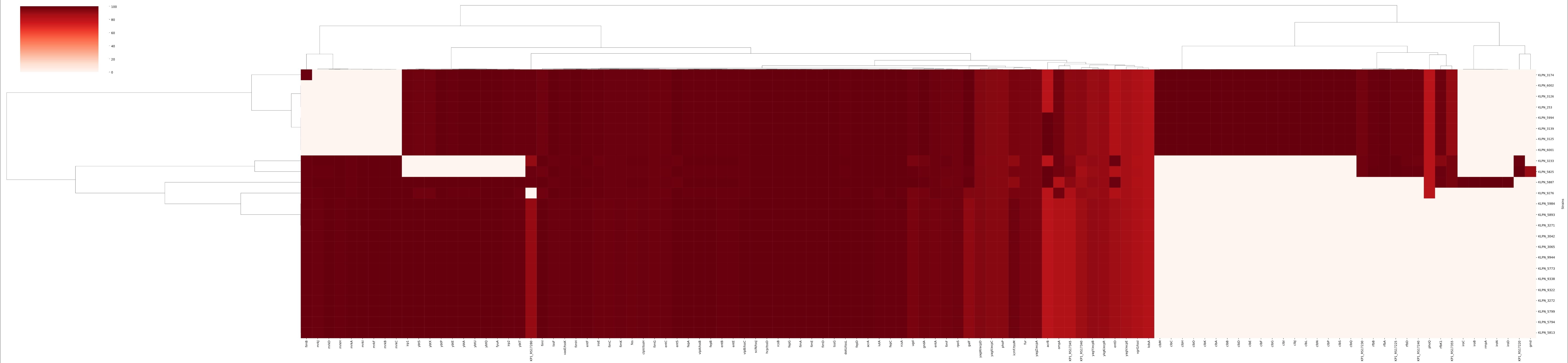

Supplement: Supplementary file 2 — Supplementary Material 2 (JPEG 472 KB) [file 42770_2026_1882_MOESM2_ESM.jpg]

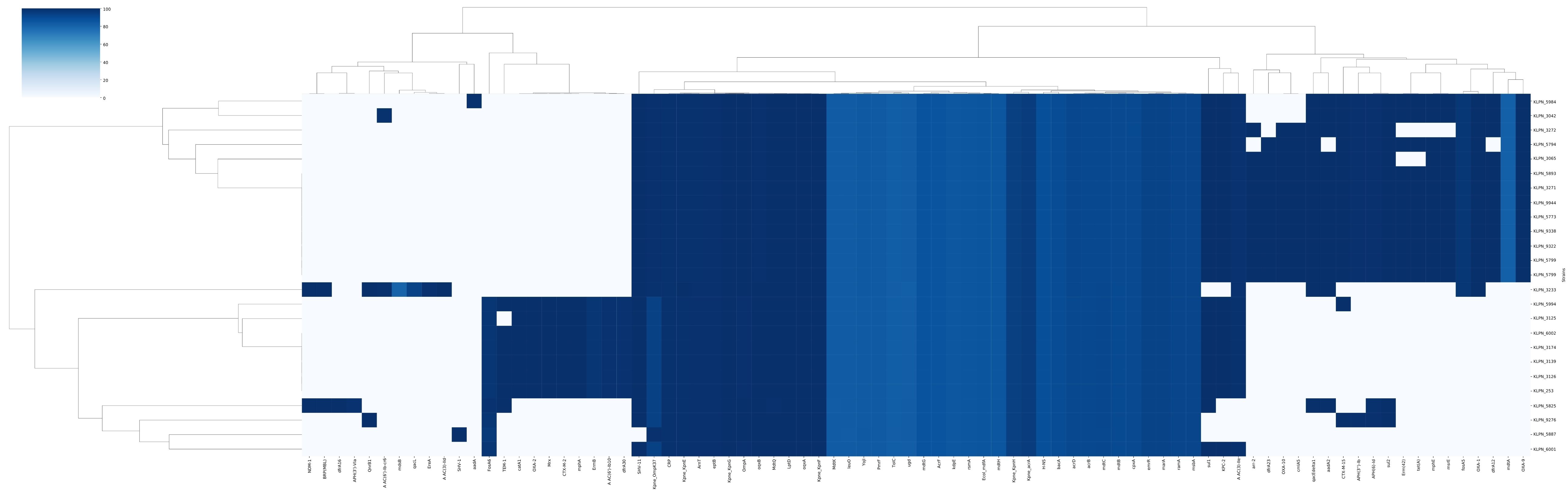

Supplement: Supplementary file 3 — Supplementary Material 3 (JPEG 364 KB) [file 42770_2026_1882_MOESM3_ESM.jpg]
