# Supplementary material for: Genomic characterization of multidrug-resistant Klebsiella pneumoniae from an outbreak in Northeastern Brazil: mechanisms of virulence and resistance
Source: Braz J Microbiol. 2026 Feb 25;57(1):62. doi: 10.1007/s42770-026-01882-3 (PMC12936292; doi:10.1007/s42770-026-01882-3)
Supplement: Supplementary file 4 — Supplementary Material 4 (DOCX 56.0 KB) [file 42770_2026_1882_MOESM4_ESM.docx]

**Genomic characterization of multidrug-resistant** *Klebsiella pneumoniae* **from an outbreak in northeastern Brazil: mechanisms of virulence and resistance**

Danillo Sales Rosa^1^ ([0000-0002-0447-5041](https://orcid.org/0000-0002-0447-5041)); Gabryel Bernardo Vieira de Lima^2^ ([0009-0000-3876-4485](https://orcid.org/0009-0000-3876-4485)); Henrique Da Silva Vieira^2^ ([0009-0008-2331-2863](https://orcid.org/0009-0008-2331-2863)), Flávia Figueira Aburjaile^2^ ([0000-0002-1067-1882](https://orcid.org/0000-0002-1067-1882)); Vasco Ariston de Carvalho Azevedo^2^ ([0000-0002-4775-2280](https://orcid.org/0000-0002-4775-2280)); Bertram Brenig^3^ ([0000-0002-7635-9656](https://orcid.org/0000-0002-7635-9656)); Mateus Matiuzzi da Costa^4^* ([0000-0002-9884-2112](https://orcid.org/0000-0002-9884-2112))

^1^Universidade Federal Rural de Pernambuco, Recife, Pernambuco 52171-900, Brazil.

^2^Universidade Federal de Minas Gerais, Belo Horizonte, Minas Gerais 31270-901, Brazil.

^3^Institute of Veterinary Medicine, University Göttingen, Göttingen 37077, Germany.

^4^Universidade Federal do Vale do São Francisco, Petrolina, Pernambuco 56304-917, Brazil.

*Corresponding author: Mateus Matiuzzi da Costa, Universidade Federal do Vale do São Francisco (UNIVASF), Petrolina, Pernambuco 56300-000, Brazil. Tel.: +55 (87) 98822-9388, e-mail: mmatiuzzicosta@gmail.com

**Supplementary table S1.** Predicted virulence genes in the genomes of *Klebsiella pneumoniae* isolates with similarity ≥80%, from the University Hospital of the Universidade Federal do Vale do São Francisco, Petrolina – PE, based on VFDB.

| **Genes** | **Number of genomes with the gene present** | **Mechanism** | **Gene complex** |
| --- | --- | --- | --- |
| *fimA* | 25 | Adherence (VFC0001) | *E. coli* type 1 fimbriae |
| *fimC* | 25 | Adherence (VFC0001) |  |
| *fimD* | 25 | Adherence (VFC0001) |  |
| *fimF* | 25 | Adherence (VFC0001) |  |
| *fimG* | 25 | Adherence (VFC0001) |  |
| *fimH* | 25 | Adherence (VFC0001) |  |
| *fimE* | 25 | Adherence (VFC0001) | *fimA* regulator |
| *fimB* | 18 | Adherence (VFC0001) | Type 1 fimbriae regulator |
| *fimI* | 25 | Adherence (VFC0001) | Required for type 1 fimbriae biosynthesis |
| *fimK* | 25 | Adherence (VFC0001) | Type 1 fimbriae regulator |
| *yagV/ecpE* | 25 | Adherence (VFC0001) | *E. coli* common pilus (ECP) |
| *yagW/ecpD* | 25 | Adherence (VFC0001) |  |
| *yagX/ecpC* | 25 | Adherence (VFC0001) |  |
| *yagY/ecpB* | 25 | Adherence (VFC0001) |  |
| *yagZ/ecpA* | 25 | Adherence (VFC0001) |  |
| *ykgK/ecpR* | 25 | Adherence (VFC0001) |  |
| *acrA* | 25 | Antimicrobial activity/Competitive advantage (VFC0325) | Periplasmic lipoprotein component of the multidrug efflux pump AcrAB-TolC in *Escherichia* *coli* and can function in chimeric constructs with three other RND family pumps, AcrD, AcrF, and YhiV |
| *acrB* | 25 | Antimicrobial activity/Competitive advantage (VFC0325) | AcrA-AcrB-TolC multidrug efflux complex (RND) |
| *mrkA* | 17 | Biofilm (VFC0271) | Type 3 fimbriae |
| *mrkB* | 17 | Biofilm (VFC0271) |  |
| *mrkC* | 17 | Biofilm (VFC0271) |  |
| *mrkD* | 17 | Biofilm (VFC0271) |  |
| *mrkF* | 17 | Biofilm (VFC0271) |  |
| *mrkH* | 17 | Biofilm (VFC0271) | Regulator and activator of type 3 fimbriae |
| *mrkI* | 17 | Biofilm (VFC0271) | Type 3 fimbriae regulator |
| *mrkJ* | 17 | Biofilm (VFC0271) | Type 3 fimbriae regulator |
| *vipA/tssB* | 25 | Effector delivery system (VFC0086) | Type VI secretion system |
| *vipB/tssC* | 25 | Effector delivery system (VFC0086) |  |
| *hcp/tssD* | 25 | Effector delivery system (VFC0086) |  |
| *tssF* | 25 | Effector delivery system (VFC0086) |  |
| *tssG* | 25 | Effector delivery system (VFC0086) |  |
| *clpV/tssH* | 25 | Effector delivery system (VFC0086) |  |
| *vgrG/tssI* | 25 | Effector delivery system (VFC0086) |  |
| *sciN/tssJ* | 25 | Effector delivery system (VFC0086) |  |
| *vasE/tssK* | 25 | Effector delivery system (VFC0086) |  |
| *dotU/tssL* | 25 | Effector delivery system (VFC0086) |  |
| *icmF/tssM* | 25 | Effector delivery system (VFC0086) |  |
| *clbA* | 8 | Exotoxin (VFC0235) | Colibactin. *pks* genomic island |
| *clbB* | 8 | Exotoxin (VFC0235) |  |
| *clbC* | 8 | Exotoxin (VFC0235) |  |
| *clbD* | 8 | Exotoxin (VFC0235) |  |
| *clbE* | 8 | Exotoxin (VFC0235) |  |
| *clbF* | 8 | Exotoxin (VFC0235) |  |
| *clbG* | 8 | Exotoxin (VFC0235) |  |
| *clbH* | 8 | Exotoxin (VFC0235) |  |
| *clbI* | 8 | Exotoxin (VFC0235) |  |
| *clbJ* | 8 | Exotoxin (VFC0235) |  |
| *clbK* | 8 | Exotoxin (VFC0235) |  |
| *clbL* | 8 | Exotoxin (VFC0235) |  |
| *clbM* | 8 | Exotoxin (VFC0235) |  |
| *clbN* | 8 | Exotoxin (VFC0235) |  |
| *clbO* | 8 | Exotoxin (VFC0235) |  |
| *clbP* | 8 | Exotoxin (VFC0235) |  |
| *clbQ* | 8 | Exotoxin (VFC0235) |  |
| *clbS* | 8 | Exotoxin (VFC0235) |  |
| *galF* | 25 | Immune modulation (VFC0258) | UTP:glucose-1-phosphate uridylyltransferase |
| *gndA* | 25 | Immune modulation (VFC0258) | NADP+-dependent 6-P-gluconate dehydrogenase |
| *gmd* | 1 | Immune modulation (VFC0258) | GDP-mannose 4,6-dehydratase |
| *kdsA* | 25 | Immune modulation (VFC0258) | 3-deoxy-8-phosphooctulonate synthase |
| *KP1_RS17220* | 2 | Immune modulation (VFC0258) | Glycosyltransferase |
| *KP1_RS17225* | 10 | Immune modulation (VFC0258) | glycosyltransferase family 4 protein |
| *KP1_RS17230* | 10 | Immune modulation (VFC0258) | Glycosyltransferase |
| *KP1_RS17240* | 25 | Immune modulation (VFC0258) | DUF4422 domain-containing protein |
| *KP1_RS17280* | 24 | Immune modulation (VFC0258) | Mannose-1-phosphate guanylyltransferase |
| *KP1_RS17340* | 10 | Immune modulation (VFC0258) | Putative capsule polysaccharide export protein precursor |
| *KP1_RS17345* | 25 | Immune modulation (VFC0258) | Capsule assembly Wzi family protein |
| *KP1_RS17355* | 11 | Immune modulation (VFC0258) | Putative acid phosphatase |
| *ompA* | 25 | Immune modulation (VFC0258) | Outer membrane protein (porin) |
| *rfbA* | 10 | Immune modulation (VFC0258) | O-antigen export ABC transporter permease |
| *rfbB* | 10 | Immune modulation (VFC0258) | O-antigen export ABC transporter ATP-binding protein |
| *rfbD* | 10 | Immune modulation (VFC0258) | UDP-galactopyranose mutase |
| *rfbK1* | 11 | Immune modulation (VFC0258) | O9 family phosphomannomutase |
| *ugd* | 25 | Immune modulation (VFC0258) | UDP-glucose 6-dehydrogenase |
| *entA* | 25 | Nutritional/Metabolic factor (VFC0272) | Enterobactin siderophore |
| *entB* | 25 | Nutritional/Metabolic factor (VFC0272) |  |
| *entC* | 25 | Nutritional/Metabolic factor (VFC0272) |  |
| *entD* | 25 | Nutritional/Metabolic factor (VFC0272) |  |
| *entE* | 25 | Nutritional/Metabolic factor (VFC0272) |  |
| *entF* | 25 | Nutritional/Metabolic factor (VFC0272) |  |
| *entS* | 25 | Nutritional/Metabolic factor (VFC0272) | Enterobactin exporter EntS |
| *fepA* | 25 | Nutritional/Metabolic factor (VFC0272) | Ferri-enterochelin permease |
| *fepB* | 25 | Nutritional/Metabolic factor (VFC0272) |  |
| *fepC* | 25 | Nutritional/Metabolic factor (VFC0272) |  |
| *fepD* | 25 | Nutritional/Metabolic factor (VFC0272) |  |
| *fepG* | 25 | Nutritional/Metabolic factor (VFC0272) |  |
| *fes* | 25 | Nutritional/Metabolic factor (VFC0272) | Enterobactin/ferric enterobactin esterase |
| *fyuA* | 23 | Nutritional/Metabolic factor (VFC0272) | siderophore yersiniabactin receptor |
| *iroB* | 1 | Nutritional/Metabolic factor (VFC0272) | Salmochelin biosynthesis C-glycosyltransferase |
| *iroC* | 1 | Nutritional/Metabolic factor (VFC0272) | Salmochelin/enterobactin export ABC transporter |
| *iroD* | 1 | Nutritional/Metabolic factor (VFC0272) | Enterochelin esterase-like protein |
| *iroE* | 25 | Nutritional/Metabolic factor (VFC0272) | Siderophore esterase |
| *iroN* | 1 | Nutritional/Metabolic factor (VFC0272) | Salmochelin receptor |
| *irp1* | 23 | Nutritional/Metabolic factor (VFC0272) | Yersiniabactin synthetase |
| *irp2* | 23 | Nutritional/Metabolic factor (VFC0272) |  |
| *ybtE* | 23 | Nutritional/Metabolic factor (VFC0272) |  |
| *ybtU* | 23 | Nutritional/Metabolic factor (VFC0272) |  |
| *ybtA* | 23 | Nutritional/Metabolic factor (VFC0272) | Yersiniabactin transcriptional regulator |
| *ybtX* | 23 | Nutritional/Metabolic factor (VFC0272) | Yersiniabactin-associated zinc MFS transporter |
| *ybtP* | 23 | Nutritional/Metabolic factor (VFC0272) | Yersiniabactin ABC transporter ATP-binding/permease protein |
| *ybtQ* | 23 | Nutritional/Metabolic factor (VFC0272) |  |
| *ybtS* | 23 | Nutritional/Metabolic factor (VFC0272) | Yersiniabactin biosynthesis salicylate synthase |
| *ybtT* | 23 | Nutritional/Metabolic factor (VFC0272) | Yersiniabactin biosynthesis thioesterase |
| *iutA* | 25 | Nutritional/Metabolic factor (VFC0272) | Ferric aerobactin receptor |
| *fur* | 25 | Regulation (VFC0301) | Ferric iron uptake transcriptional regulator |
| *phoP* | 25 | Regulation (VFC0301) | Two-Component Regulatory System PhoP-PhoQ |
| *phoQ* | 2 | Regulation (VFC0301) |  |
| *rcsA* | 25 | Regulation (VFC0301) | Transcriptional regulator |
| *rcsB* | 25 | Regulation (VFC0301) | Response regulator transcription factor |
| *rmpA* | 1 | Regulation (VFC0301) | Regulator of mucoid phenotype A |
| *rpoS* | 25 | Regulation (VFC0301) | RNA polymerase sigma factor |

**Supplementary table S2.** Predicted resistance genes in the genomes of *Klebsiella pneumoniae* isolates with similarity ≥80%, from the University Hospital of the Universidade Federal do Vale do São Francisco, Petrolina – PE, based on CARD.

| **Genes** | **Number of genomes with the gene present** | **Mechanism** | **Gene complex** |
| --- | --- | --- | --- |
| *Kpne_acrA* | 25 | antibiotic efflux | AcrA-AcrB-TolC multidrug efflux complex (RND) |
| *acrB* | 25 | antibiotic efflux |  |
| *TolC* | 25 | antibiotic efflux |  |
| *acrD* | 25 | antibiotic efflux | Multidrug efflux pump RND permease AcrB. The gene acrD is paralogous to acrB |
| *AcrF* | 25 | antibiotic efflux | Multidrug efflux pump RND permease AcrF. Is highly homologous to the AcrAB multidrug efflux system |
| *cmlA5* | 11 | antibiotic efflux | Chloramphenicol efflux MFS transporter CmlA5 |
| *cpxA* | 25 | antibiotic efflux | Sensor histidine kinase CpxA. The sensor protein CpxA is part of the two-component CpxA/CpxR signal transduction system (RND) |
| *CRP* | 25 | antibiotic efflux | cAMP-activated global transcriptional regulator CRP (RND) |
| *Ecol_mdfA* | 25 | antibiotic efflux | Multidrug efflux pump MdfA/Na(+):H(+) antiporter/K(+):H(+) antiporter (MFS) |
| *emrR* | 25 | antibiotic efflux | Transcriptional repressor of the emrAB operon (MFS) |
| *H-NS* | 25 | antibiotic efflux | H-NS is a histone-like protein involved in global gene regulation (RND) |
| *kdpE* | 25 | antibiotic efflux | transcriptional activator that is part of the two-component system KdpD/KdpE |
| *Kpne_KpnE* | 25 | antibiotic efflux | KpnEF efflux pump (SMR) |
| *Kpne_KpnF* | 25 | antibiotic efflux |  |
| *Kpne_KpnG* | 25 | antibiotic efflux | KpnGH-TolC efflux pump (MFS) |
| *Kpne_KpnH* | 25 | antibiotic efflux |  |
| *TolC* | 25 | antibiotic efflux |  |
| *leuO* | 25 | antibiotic efflux | DNA-binding transcriptional dual regulator LeuO (MFS) |
| *LptD* | 25 | antibiotic efflux | Involved in LPS transport in a ABC Transporter efflux system |
| *mdsB* | 2 | antibiotic efflux | The inner membrane transporter of the multidrug and metal efflux complex MdsABC (RND) |
| *mdtA* | 14 | antibiotic efflux | MdtABC-TolC is a multidrug efflux (RND) |
| *mdtB* | 25 | antibiotic efflux |  |
| *mdtC* | 25 | antibiotic efflux |  |
| *TolC* | 25 | antibiotic efflux |  |
| *mdtG* | 25 | antibiotic efflux | Efflux pump MdtG (MFS) |
| *mdtH* | 25 | antibiotic efflux | Multidrug efflux pump MdtH (MFS) |
| *MdtK* | 25 | antibiotic efflux | Multidrug efflux pump MdtK (MATE) |
| *msbA* | 25 | antibiotic efflux | ATP-binding lipopolysaccharide transport protein (ABC) |
| *oqxA* | 25 | antibiotic efflux | Multidrug efflux RND transporter periplasmic adaptor |
| *oqxB* | 25 | antibiotic efflux |  |
| *qacEdelta1* | 15 | antibiotic efflux | Multidrug efflux MFS |
| *qacL* | 2 | antibiotic efflux | Subunit of the qac multidrug efflux pump (SMR) |
| *rsmA* | 25 | antibiotic efflux | Its negative effect on MexEF-OprN overexpression has been noted to confer resistance to various antibiotics. (RND) |
| *tet(A)* | 11 | antibiotic efflux | Tet(A)/Tet(B)/Tet(C) family tetracycline efflux MFS transporter |
| *TolC* | 25 | antibiotic efflux | Outer membrane channel TolC (RND) |
| *YojI* | 25 | antibiotic efflux | ABC transporter family protein/microcin J25 efflux protein |
| *AAC(3)-IId* | 1 | antibiotic inactivation | Aminoglycoside N-acetyltransferase |
| *AAC(3)-IIe* | 22 | antibiotic inactivation | Aminoglycoside N-acetyltransferase |
| *AAC(6')-Ib10* | 7 | antibiotic inactivation | Integron-encoded aminoglycoside acetyltransferase |
| *AAC(6')-Ib-cr6* | 2 | antibiotic inactivation | Fluoroquinolone-acetylating aminoglycoside acetyltransferase variant |
| *aadA* | 1 | antibiotic inactivation | Aminoglycoside adenyltransferase |
| *aadA2* | 13 | antibiotic inactivation | Aminoglycoside nucleotidyltransferase |
| *APH(3'')-Ib* | 15 | antibiotic inactivation | Aminoglycoside O-phosphotransferase APH(3'')-Ib |
| *APH(6)-Id* | 15 | antibiotic inactivation | Aminoglycoside O-phosphotransferase APH(6)-Id |
| *APH(3')-VIa* | 1 | antibiotic inactivation | Aminoglycoside phosphotransferase APH(3')-VIa |
| *arr-2* | 11 | antibiotic inactivation | Integron-encoded ribosyltransferase |
| *BRP(MBL)* | 1 | antibiotic inactivation | Bleomycin resistant protein |
| *catA1* | 7 | antibiotic inactivation | Chloramphenicol acetyltransferase (CAT) |
| *CTX-M-15* | 15 | antibiotic inactivation | CTX-M beta-lactamase |
| *CTX-M-2* | 7 | antibiotic inactivation | CTX-M beta-lactamase |
| *EreA* | 1 | antibiotic inactivation | Macrolide esterase |
| *fosA5* | 14 | antibiotic inactivation | Fosfomycin thiol transferase |
| *FosA6* | 10 | antibiotic inactivation | Fosfomycin thiol transferase |
| *KPC-2* | 22 | antibiotic inactivation | Carbapenem-hydrolyzing beta-lactamase KPC-2 |
| *mphA* | 7 | antibiotic inactivation | Macrolide phosphotransferase (MPH) |
| *mphE* | 12 | antibiotic inactivation | Macrolide phosphotransferase (MPH) |
| *Mrx* | 7 | antibiotic inactivation | Macrolide phosphotransferase (MPH) |
| *NDM-1* | 1 | antibiotic inactivation | NDM beta-lactamase |
| *OXA-1* | 14 | antibiotic inactivation | OXA beta-lactamase |
| *OXA-10* | 11 | antibiotic inactivation | OXA beta-lactamase |
| *OXA-2* | 7 | antibiotic inactivation | OXA beta-lactamase |
| *OXA-9* | 13 | antibiotic inactivation | OXA beta-lactamase |
| *SHV-1* | 1 | antibiotic inactivation | SHV beta-lactamase |
| *SHV-11* | 24 | antibiotic inactivation | SHV beta-lactamase |
| *TEM-1* | 7 | antibiotic inactivation | TEM beta-lactamase |
| *ArnT* | 25 | antibiotic target alteration | Pmr phosphoethanolamine transferase |
| *bacA* | 25 | antibiotic target alteration | Undecaprenyl pyrophosphate related proteins |
| *eptB* | 25 | antibiotic target alteration | Pmr phosphoethanolamine transferase |
| *Erm(42)* | 11 | antibiotic target alteration | Erm 23S ribosomal RNA methyltransferase |
| *ErmB* | 7 | antibiotic target alteration | Erm 23S ribosomal RNA methyltransferase |
| *PmrF* | 25 | antibiotic target alteration | Pmr phosphoethanolamine transferase |
| *ugd* | 25 | antibiotic target alteration | Pmr phosphoethanolamine transferase |
| *msrE* | 12 | antibiotic target protection | Msr-type ABC-F protein |
| *QnrB1* | 2 | antibiotic target protection | Quinolone resistance protein (qnr) |
| *dfrA12* | 12 | antibiotic target replacement | Trimethoprim resistant dihydrofolate reductase dfr |
| *dfrA16* | 1 | antibiotic target replacement | Trimethoprim resistant dihydrofolate reductase dfr |
| *dfrA23* | 10 | antibiotic target replacement | Trimethoprim resistant dihydrofolate reductase dfr |
| *dfrA30* | 7 | antibiotic target replacement | Trimethoprim resistant dihydrofolate reductase dfr |
| *sul1* | 21 | antibiotic target replacement | Sulfonamide resistant sul |
| *sul2* | 14 | antibiotic target replacement | Sulfonamide resistant sul |
| *Kpne_OmpK37* | 25 | reduced permeability to antibiotic | General Bacterial Porin with reduced permeability to beta-lactams |
| *Mdtq* | 25 | reduced permeability to antibiotic | Outer Membrane Porin (Opr) |
| *OmpA* | 25 | reduced permeability to antibiotic | General Bacterial Porin with reduced permeability to peptide antibiotics |
| *marA* | 25 | reduced permeability to antibiotic, antibiotic efflux | Resistance-nodulation-cell division (RND) antibiotic efflux pump, General Bacterial Porin with reduced permeability to beta-lactams |
| *ramA* | 25 | reduced permeability to antibiotic, antibiotic efflux | General Bacterial Porin with reduced permeability to beta-lactams, resistance-nodulation-cell division (RND) antibiotic efflux pump |

**Tabela suplementar S3.** Detailed background information and results of Multilocus Sequence Typing analyses of *Klebsiella pneumoniae* isolates, from the University Hospital of the Universidade Federal do Vale do São Francisco, Petrolina – PE.

| **Isolate** | **ST** | ***gapA*** | ***infB*** | ***mdh*** | ***pgi*** | ***phoE*** | ***rpoB*** | ***tonB*** |
| --- | --- | --- | --- | --- | --- | --- | --- | --- |
| KLPN_253 | 11 | 3 | 3 | 1 | 1 | 1 | 1 | 4 |
| KLPN_6002 | 11 | 3 | 3 | 1 | 1 | 1 | 1 | 4 |
| KLPN_3125 | 11 | 3 | 3 | 1 | 1 | 1 | 1 | 4 |
| KLPN_5994 | 11 | 3 | 3 | 1 | 1 | 1 | 1 | 4 |
| KLPN_3126 | 11 | 3 | 3 | 1 | 1 | 1 | 1 | 4 |
| KLPN_9273 | 11 | 3 | 3 | 1 | 1 | 1 | 1 | 4 |
| KLPN_3139 | 11 | 3 | 3 | 1 | 1 | 1 | 1 | 4 |
| KLPN_3174 | 11 | 3 | 3 | 1 | 1 | 1 | 1 | 4 |
| KLPN_6001 | 11 | 3 | 3 | 1 | 1 | 1 | 1 | 4 |
| KLPN_5813 | 273 | 3 | 3 | 1 | 1 | 1 | 1 | 4 |
| KLPN_3042 | 273 | 3 | 4 | 6 | 1 | 7 | 4 | 4 |
| KLPN_9944 | 273 | 3 | 4 | 6 | 1 | 7 | 4 | 4 |
| KLPN_5984 | 273 | 3 | 4 | 6 | 1 | 7 | 4 | 4 |
| KLPN_5773 | 273 | 3 | 4 | 6 | 1 | 7 | 4 | 4 |
| KLPN_5794 | 273 | 3 | 4 | 6 | 1 | 7 | 4 | 4 |
| KLPN_5799 | 273 | 3 | 4 | 6 | 1 | 7 | 4 | 4 |
| KLPN_3065 | 273 | 3 | 4 | 6 | 1 | 7 | 4 | 4 |
| KLPN_5893 | 273 | 3 | 4 | 6 | 1 | 7 | 4 | 4 |
| KLPN_9322 | 273 | 3 | 4 | 6 | 1 | 7 | 4 | 4 |
| KLPN_9338 | 273 | 3 | 4 | 6 | 1 | 7 | 4 | 4 |
| KLPN_3272 | 273 | 3 | 4 | 6 | 1 | 7 | 4 | 4 |
| KLPN_3271 | 273 | 3 | 4 | 6 | 1 | 7 | 4 | 4 |
| KLPN_5825 | 395 | 3 | 4 | 6 | 1 | 7 | 4 | 4 |
| KLPN_5887 | 636 | 3 | 1 | 2 | 4 | 1 | 1 | 4 |
| KLPN_3233 | 5209 | 2 | 5 | 1 | 1 | 4 | 1 | 4 |

ST: sequence typing
